# RNA-SEQ Reveals Transcriptional Level Changes of Poplar Roots in Different Forms of Nitrogen Treatments

**DOI:** 10.3389/fpls.2016.00051

**Published:** 2016-02-02

**Authors:** Chun-Pu Qu, Zhi-Ru Xu, Yan-Bo Hu, Yao Lu, Cheng-Jun Yang, Guang-Yu Sun, Guan-Jun Liu

**Affiliations:** ^1^State Key Laboratory of Tree Genetics and Breeding, School of Forestry, Northeast Forestry UniversityHarbin, China; ^2^College of Life Science, Northeast Forestry UniversityHarbin, China; ^3^School of Forestry, Northeast Forestry UniversityHarbin, China

**Keywords:** poplar, nitrogen metabolism, RNA-SEQ, nitrogen forms, long-time nitrogen treatment

## Abstract

Poplar has emerged as a model plant for better understanding cellular and molecular changes accompanying tree growth, development, and response to environment. Long-term application of different forms of nitrogen (such as ${\text{NO}}_{3}^{-}$-N and ${\text{NH}}_{4}^{+}$-N) may cause morphological changes of poplar roots; however, the molecular level changes are still not well-known. In this study, we analyzed the expression profiling of poplar roots treated by three forms of nitrogen: S1 (${\text{NH}}_{4}^{+}$), S2 (NH_4_NO_3_), and S3 (${\text{NO}}_{3}^{-}$) by using RNA-SEQ technique. We found 463 genes significantly differentially expressed in roots by different N treatments, of which a total of 112 genes were found to differentially express between S1 and S2, 171 genes between S2 and S3, and 319 genes between S1 and S3. A cluster analysis shows significant difference in many transcription factor families and functional genes family under different N forms. Through an analysis of Mapman metabolic pathway, we found that the significantly differentially expressed genes are associated with fermentation, glycolysis, and tricarboxylic acid cycle (TCA), secondary metabolism, hormone metabolism, and transport processing. Interestingly, we did not find significantly differentially expressed genes in N metabolism pathway, mitochondrial electron transport/ATP synthesis and mineral nutrition. We also found abundant candidate genes (20 transcription factors and 30 functional genes) regulating morphology changes of poplar roots under the three N forms. The results obtained are beneficial to a better understanding of the potential molecular and cellular mechanisms regulating root morphology changes under different N treatments.

## Introduction

Nitrogen (N) element is one of macronutrients essential for plant growth, which accounts for 1.5–2% of plant dry matter and ~16% of total plant protein (Frink et al., 1999). Plant roots mainly take up inorganic nitrogen in the form of ammonium (${\text{NH}}_{4}^{+}$-N) and/or nitrate (${\text{NO}}_{3}^{-}$-N) from soil. For most plants, a mixed nutrition of ${\text{NO}}_{3}^{-}$ and ${\text{NH}}_{4}^{+}$ is superior over sole ${\text{NH}}_{4}^{+}$-N or ${\text{NO}}_{3}^{-}$-N source (Marschner, 2011). The proportion of ${\text{NO}}_{3}^{-}$ to ${\text{NH}}_{4}^{+}$ for optimal plant growth depends on plant species, developmental stage, environmental conditions, and the total concentrations of supplied N (Jackson and Caldwell, 1993; Luo et al., 2013a; Zhang et al., 2014). Morphological characters of plant roots and shoots are significantly different when it is supplied by a moderate concentration of ${\text{NO}}_{3}^{-}$ and ${\text{NH}}_{4}^{+}$, respectively, as sole N source (Schortemeyer et al., 1997; Claussen, 2002; Wang et al., 2003). For example, total dry weight of tomato plants was decreased by 32–86% when it was cultured by ${\text{NH}}_{4}^{+}$ as sole N source, both total dry weight and fruit dry weight were increased by 11 and 30% when adding a low concentration of ${\text{NO}}_{3}^{-}$ (N:A ratio = 75:25) to the culture solution (Wang et al., 2003). ${\text{NH}}_{4}^{+}$ as the sole N source resulted in lower dry weight of tobacco roots as compared with the other N forms (${\text{NO}}_{3}^{-}$ and NH_4_NO_3_; Zou et al., 2005). Moreover, there were significant differences in physiological characters, including activity of glutamate dehydrogenase (*Acer pseudoplatanus*), total amino acid concentration (soybeans), photosynthetic rates (wheat and maize), phosphoenolpyruvate carboxylase (Alfalfa), glutamine synthetase (Pea), type II NAD(P)H dehydrogenase, AOX genes and proline oxidase (Arabidopsis) when the plants were supplied with ${\text{NH}}_{4}^{+}$ and ${\text{NO}}_{3}^{-}$, respectively (Goodchild and Givan, 1990; Chaillou et al., 1991; Cramer and Lewis, 1993; Pasqualini et al., 2001; Frechilla et al., 2002; Escobar et al., 2006; Patterson et al., 2010).

Morphological and physiological changes induced by different N forms are closely linked to transcription-level changes. In recent years, the role of ${\text{NO}}_{3}^{-}$ in a global regulation of plant transcriptome has been extensively explored. The previous studies show that as compared with N-free samples, supplying ${\text{NO}}_{3}^{-}$ to *Arabidopsis* seedlings make transcriptional-level changes of the biological processes including transcription and RNA processing, biosynthesis of amino acids and nucleic acids, trehalose metabolism, hormone biosynthesis, and N assimilation as well as *PtaNAC1*, a transcription factor, which is thought to be associated with root architecture under low nitrogen (LN) conditions (Wang et al., 2000, 2003; Scheible et al., 2004; Bi et al., 2007; Gifford et al., 2008; Wei et al., 2013). Researches on molecular effects of ${\text{NH}}_{4}^{+}$ and/or NH_4_NO_3_ nutrition are less compared with the studies on that of ${\text{NO}}_{3}^{-}$, though several recent publications reported regulation of ${\text{NH}}_{4}^{+}$ on gene expression in various plant systems. Fizames et al. (2004) identified 270 genes differentially expressed in *Arabidopsis* roots when supplied with ${\text{NO}}_{3}^{-}$ or NH_4_NO_3_ as N source. Zhu et al. (2006) demonstrated that ${\text{NH}}_{4}^{+}$ as N source stimulated sulfur assimilation in rice leaves. In alfalfa, Ruffel et al. (2008) revealed that over 3000 genes expression was regulated by the status of plant N supply. Poplar has emerged as a model system for understanding molecular mechanisms of woody plants growth, development, and response to environment (Brunner et al., 2004). Some progresses have been achieved in morphological, physiological characteristics of some fast-growing poplar trees (such as *P. simonii* × *P. nigra*) and selection of stress-tolerant genes (Wang et al., 2011; Chen et al., 2012; Li et al., 2012; Luo et al., 2013a, 2015; Gan et al., 2015). However, to date, few studies focus on linkages of morphological changes of roots and transcriptional-level characteristics when poplar plants were treated by different N-forms nutrition (${\text{NH}}_{4}^{+}$or ${\text{NO}}_{3}^{-}$, or both). In this study, we hypothesized there were potential coupling changes of N metabolism-related genes and root morphology when the poplar plants were treated by different N forms. So we examined transcriptome profiling of the *P. simonii* × *P. nigra* roots using high throughput sequencing technique and analyzed potential effects of long-term different N forms on N metabolism and root morphology-related genes of hydroponic-cultured *P. simonii* × *P. nigra* seedlings by a large-scale comparative transcriptomes analysis.

## Materials and methods

### Plant material and treatments

**Figure 1:**
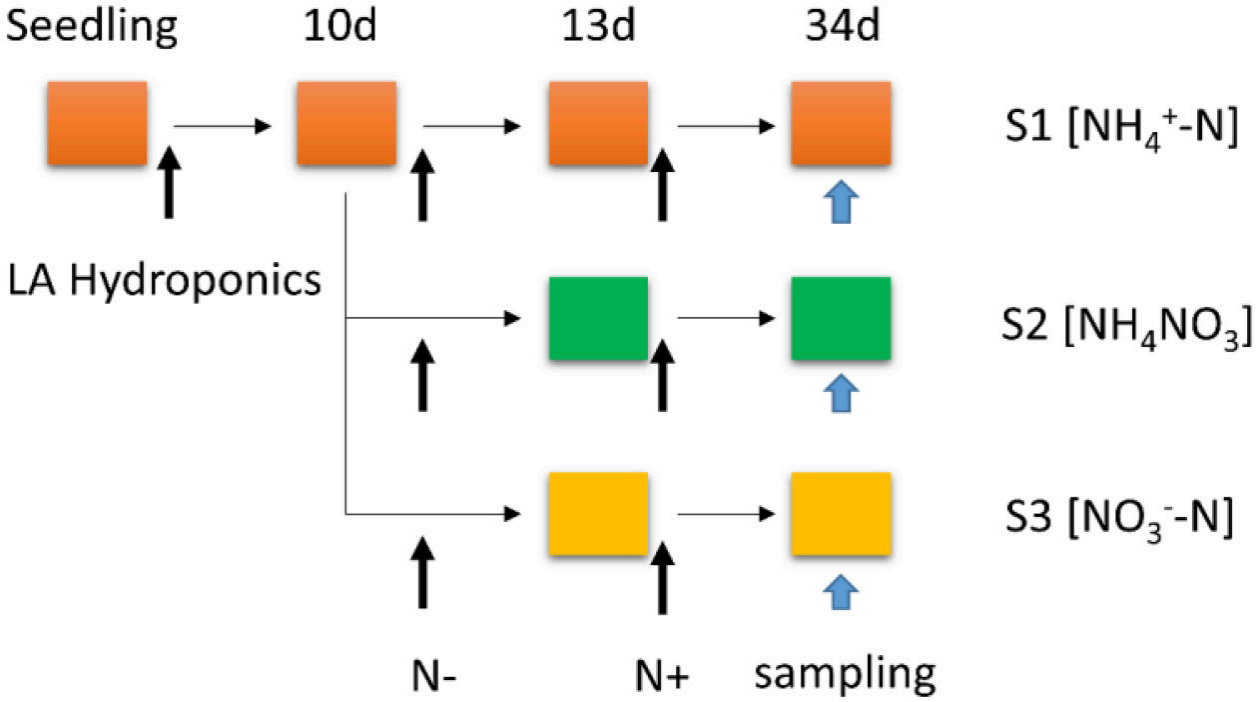
**Schematic representation of the experimental set-up: poplar plants were grown hydroponically in complete LA liquid medium (N^+^ medium) for 10 days, then transferred to nitrogen-free medium (N^−^ medium) for 3 days; subsets of plants were transferred back to complete medium (N resupply, S1 [${\text{NH}}_{4}^{+}$-N]; S2 [NH_4_NO_3_]; S3 [${\text{NO}}_{3}^{-}$-N]) for another 21 days**. The total treatment period is 34 days.

Poplar seedlings (*P. simonii* × *P. nigra*) were germinated on LA media (Hewitt, 1966) with 2% (w/v) sucrose and grown in a growth chamber (light intensity of 200 μmol photons m^−2^ s^−1^ for 16 h per day, day/night temperature of 24/22°C, relative humidity of 50–55%) for 25 days. Then the seedlings were transferred to a 4.0-L LA nutrient medium for 10 days, containing complete nutrient solution within hydroponic boxes under a 16/8 h light/dark regime at 24/22°C and constant (60–65%) relative humidity. The seedlings were transferred to N-free medium for 3 days growth, which the time of N-free treatment was determined according to the result of Balazadeh et al. (2014) and our preliminary test (data not shown), then subsets of seedlings were transferred back to LA complete mediums containing 1 mM ${\text{NH}}_{4}^{+}$, 0.5 mM NH_4_NO_3_, or 1 mM ${\text{NO}}_{3}^{-}$, respectively. The different N forms (S1 [${\text{NH}}_{4}^{+}$-N]; S2 [NH_4_NO_3_]; S3 [${\text{NO}}_{3}^{-}$-N]) were resupplied for another 21 days. To minimize the effect of the altered N content on osmotic potential, the nutrient solution was augmented by a certain amount of sodium chloride to maintain the same cation concentration in the nutrient medium. Samples were taken from the roots with different N^+^ processing for 21 days (Urbanczyk and Fernie, 2005; Wei et al., 2013). Then samples were stored at −80°C for RNA extraction (Figure 1).

### Root measurements

Root length (length of taproots from stem end to root tip) was measured using a ruler (Figure S1). Subsequently, the samples were ground into fine powder in liquid N with a mortar and pestle. Frozen powder of each sample was dried at 60°C for 24 h, then the dry weight was calculated.

### Illumina sequencing and data processing

Total RNA was isolated by using a modified CTAB method (Chen et al., 2009; Qu et al., 2010), then sent to Beijing Genomics Institute (Shenzhen) where the libraries were produced and sequenced using the Illumina Genome Analyzer (Solexa). All the samples for Digital Gene Expression were run in two biological replicates, which each replicate is a mixture of root samples from four individual seedlings. Sequence tag preparation was done with Illumina's Digital Gene Expression Tag Profiling Kit according to the manufacturer's protocol (version 2.1B). Firstly, the total RNA samples were treated with DNase I to degrade any possible DNA contamination. Then the mRNA was enriched by using the oligo (dT) magnetic beads (for eukaryotes). After mixed with the fragmentation buffer, the mRNA was fragmented into short fragments (about 200 bp). Then the double strands of cDNA were synthesized by a series of primers, buffer, RNase H and DNA polymerase I. The double strand cDNA was purified with magnetic beads. End reparation and 3′-end single nucleotide A (adenine) addition was then performed. Finally, the fragments were ligated with the adaptors and enriched by PCR amplification, each fragment will generate millions of raw reads. Raw sequences were transformed into clean reads after certain steps of data processing, including removal of the 3′ adaptor sequence, empty reads, and low-quality reads.

All clean reads were mapped to the *poplar* × *trichocarpa Torr. Gray* contigs assembly using SOAP2 and only no more than a 2-nucleotide mismatch was allowed (Li et al., 2009). Clean reads mapped to the reference contigs assembly from multiple genes were filtered. The remaining clean reads were designed as unambiguous clean reads. The number of unambiguous clean reads for each gene was calculated and then normalized to RPKM (Reads Per Kb per Million reads), which associated the read number with gene expression levels (Morrissy et al., 2009). Differential gene expression between different nitrogen forms samples was determined by taking the log_2_ ratio of RPKM.

### Identification of differentially expressed genes and gene ontology

The NOIseq was used to identify differentially expressed genes for the samples treated by different N forms. Probability ≥ 0.8 and the absolute value of log_2_ Ratio > 1 were used as the threshold to judge the significance of gene expression difference (Tarazona et al., 2011). Cluster analysis of gene expression patterns was performed by Genesis based on the K-means method (Soukas et al., 2000; de Hoon et al., 2004). Gene ontology (GO) analysis was applied to predict gene function and calculate the functional category distribution frequency (Du et al., 2010). Pathway analysis was mainly based on the Mapman (Thimm et al., 2004).

### Data validation by qRT-PCR

The primers used for qRT-PCR validation are listed in Table S1. They were designed on the basis of poplar refseq mRNA sequences using the Primer-BLAST web resource at NCBI (National Center for Biotechnology Information; http://www.ncbi.nlm.nih.gov/BLAST). Quantitative RT-PCR (qRT-PCR) was performed using the ABI7500 Real Time System (Applied Biosystems). Gene expression was analyzed quantitatively using the SYBR Green detection system with melting curve. Amplification conditions were 95°C for 3 min, followed by 40 cycles of: denaturation, 95°C for 15 s; annealing (55–60°C) for 20 s; extension at 72°C for 34 s. Samples for qRT-PCR were run in three biological replicates and two technical replicates. The results were normalized using the Pfaffl method to report relative expression (Pfaffl, 2001). For normalization of gene expression, *CYC063* and *UBQ7* were used as internal standard (Figure S2).

### Statistical analysis of root morphological parameters

All data were analyzed using SPSS 19.0 software (SPSS, Inc., Chicago, IL, USA). The root length and dry weight of poplar seedlings with different N forms were compared by one-way ANOVA on the basis of Duncan's test at the significance level of 0.05 (*P* < 0.05).

## Results

### Morphological characters of poplar roots under different N forms

**Figure 2:**
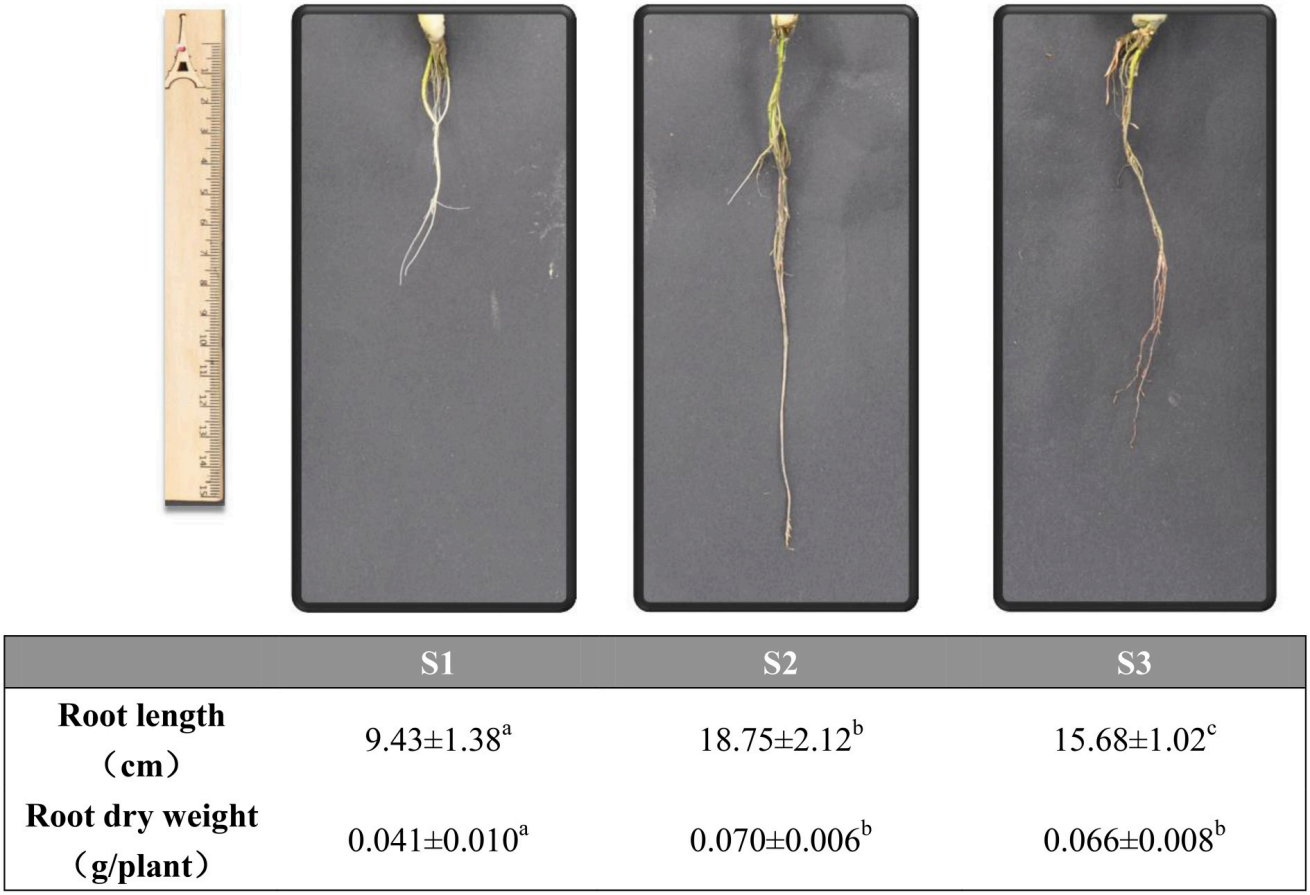
**Morphological parameters of poplar roots with different N forms for 21 days**. **Upper:** The picture of the root under different N forms; **Lower:** Length of roots and the dry weighs of roots. Values are the mean of four replicates ± SE. a, b, and c indicate significant difference based on ANOVA analysis and Duncan's test (*P* < 0.05).

Significant difference in root length and dry weight was found in roots treated by different N forms for 21 days (Figure 2). Root length and dry weight of ${\text{NO}}_{3}^{-}$ and NH_4_NO_3_ treated seedlings were higher than that of ${\text{NH}}_{4}^{+}$ treated seedlings for 21 days.

### Illumina sequencing and aligning to the reference genome

We sequenced three groups of cDNA libraries, S1 (${\text{NH}}_{4}^{+}$), S2 (NH_4_NO_3_), and S3 (${\text{NO}}_{3}^{-}$), and generated 18,232,002 sequence reads, encompassing 1.71 Gb of sequence data (Table 1). Each treatment was represented by ~6 million reads that were sufficient for the quantitative analysis of gene expression. The sequence reads were aligned to the poplar reference genome database using soap2 software (set to allow two base mismatches). Of the total reads, 71.39% were matched either to a unique (62.50%) or multiple (8.88%) genomic locations; the remaining 28.61% were unmatched (Table 1). Only the reads aligning entirely inside exonic regions were matched, the reads from exon-exon junction regions were not matched.

**Table 1:** **Summary of read numbers based on the RNA-SEQ data from poplar roots under different N forms**.

	**S1 [${\text{NH}}_{4}^{+}$]**	**S2 [NH_4_NO_3_]**	**S3 [${\text{NO}}_{3}^{-}$]**
Total reads	5,981,660	6,075,136	6,175,206
Mapped reads	3,829,967	4,503,472	4,694,482
	(64.04%)	(74.10%)	(76.03%)
Unique match	3,226,026	3,996,757	4,186,976
	(53.94%)	(65.76%)	(67.81%)
Multi-position match	603,940	506,714	507,506
	(10.08%)	(8.34%)	(8.22%)
Unmapped reads	2,151,693	1,571,664	1,480,724
	(35.96%)	(25.90%)	(23.97%)

### Global analysis of gene expression

A total of 22,414, 25,691, and 26,170 genes, ranging from 100 to ≥2000 bp, were detected in the samples of S1, S2, and S3, respectively. As shown in Table 2, the proportion of sequences with matches to poplar databases was higher among the longer assembled sequences. Specifically, a match efficiency of 32.23% was observed for sequences longer than 2000 bp, whereas the match efficiency decreased to 16.89% for those ranging from 500 to 1000 bp, and to 5.62% for sequences between 100 and 500 bp (Table 2).

**Table 2:** **Distribution of the gene sequences detected in poplar roots treated by different forms of nitrogen by RNA-SEQ**.

**Gene length (bp)**	**Total number**	**Percentage (%)**
100–500	2268	5.62
500–1000	6817	16.89
1000–1500	9553	23.67
1500–2000	8715	21.59
≥2000	13,008	32.23
Total	40,361	100

### Gene expression profiles under different nitrogen treatments

**Figure 3:**
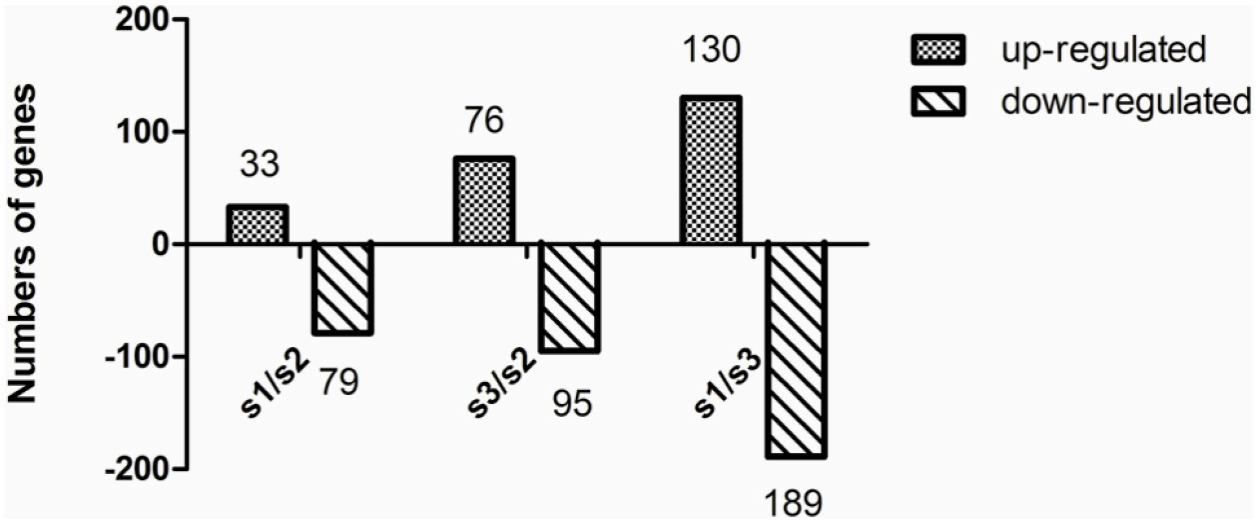
**Change in gene expression profiles among different N forms**. S1, ${\text{NH}}_{4}^{+}$ treatment; S2, NH_4_NO_3_; S3, ${\text{NO}}_{3}^{-}$. The number of up-regulated and down-regulated genes between S1 and S2, and between S2 and S3 are summarized. Between the S1 and S2 libraries, there are 33 up-regulated genes and 79 down-regulated genes, while 76 up-regulated genes and 95 down-regulated genes between the S3 and S2 libraries, and 130 up-regulated genes and 189 down-regulated genes between the S1 and S3 libraries.

To obtain statistical differences in gene expression among different N treated libraries, we compared the RPKM-derived read count using a likelihood ratio test. To minimize false positives and negatives, we assumed that a statistical analysis was reliable when applied to genes with an RPKM value ≥ 2 in both of the two replication libraries. It should be noted that the statistical significances are based on expected sampling distributions. To determine the differentially expressed genes among different N treated libraries, the threshold we used is a two-fold or greater change in expression and Probability ≥ 0.8, and we obtained a set of 602 DEGs (Tables S2–S4). A total of 112 significantly changed genes were detected between the S1 and S2 libraries, with 33 up-regulated genes and 79 down-regulated genes (Figure 3; Table S3). Between the S2 and S3 libraries, a total of 171 DEGs were detected, with 76 up-regulated genes and 95 down-regulated genes (Figure 3; Table S4). This suggests that the differentially expressed genes between S1/S2 is smaller than that between S3/S2. After eliminating duplicate genes, we found 463 genes significantly differentially expressed between the N treatments. All the predicted poplar genes were assigned to different functional categories using Blast2GO (version 2.2.5; http://www.blast2go.org/; Conesa et al., 2005).

### Functional analysis of DEGs based on RNA-SEQ data

Based on sequence homology, all the significantly changed genes were categorized into 26 functional groups in the three main categories (cellular component, molecular function, and biological process) of the GO classification. Among the groups, nine functional groups were significantly enriched (*P* ≤ 0.05), including extra cellular region (GO:0005576), oxidoreductase activity (GO:0016491), dioxygenase activity (GO:0051213), oxidoreductase activity (single donors; GO:0016701), oxidoreductase activity (paired donors; GO:0016705), ion binding (GO:0043167), cation binding (GO:0043169), plant-type cell wall modification (GO:0009827), and cell wall modification (GO:0042545; Table 3).

**Table 3:** **Summary of significantly over-represented functional groups in ammonium-regulated and nitrate-regulated gene sets**.

**Nitrogen conditions**	**Category**	**Gene ontology term**	**Observed frequency (%)**	**Expected frequency (%)**	**Corrected *P*-value**
S2-vs.-S1	C	Extracellular region	15.40	2.30	0.04456
	F	Oxidoreductase activity	36.70	11.90	0.00026
		Dioxygenase activity	10.20	0.60	0.00053
		Oxidoreductase activity (single donors)	10.20	0.70	0.00106
		Oxidoreductase activity (paired donors)	8.20	0.40	0.00231
		Ion binding	42.90	21.90	0.03417
		Cation binding	40.80	20.30	0.03473
S2-vs.-S3	P	Plant-type cell wall modification	6.30	0.10	3.51E-05
		Cell wall modification	7.90	0.20	5.29E-05

### Clustering of DEGs in the three N-treated conditions

**Figure 4:**
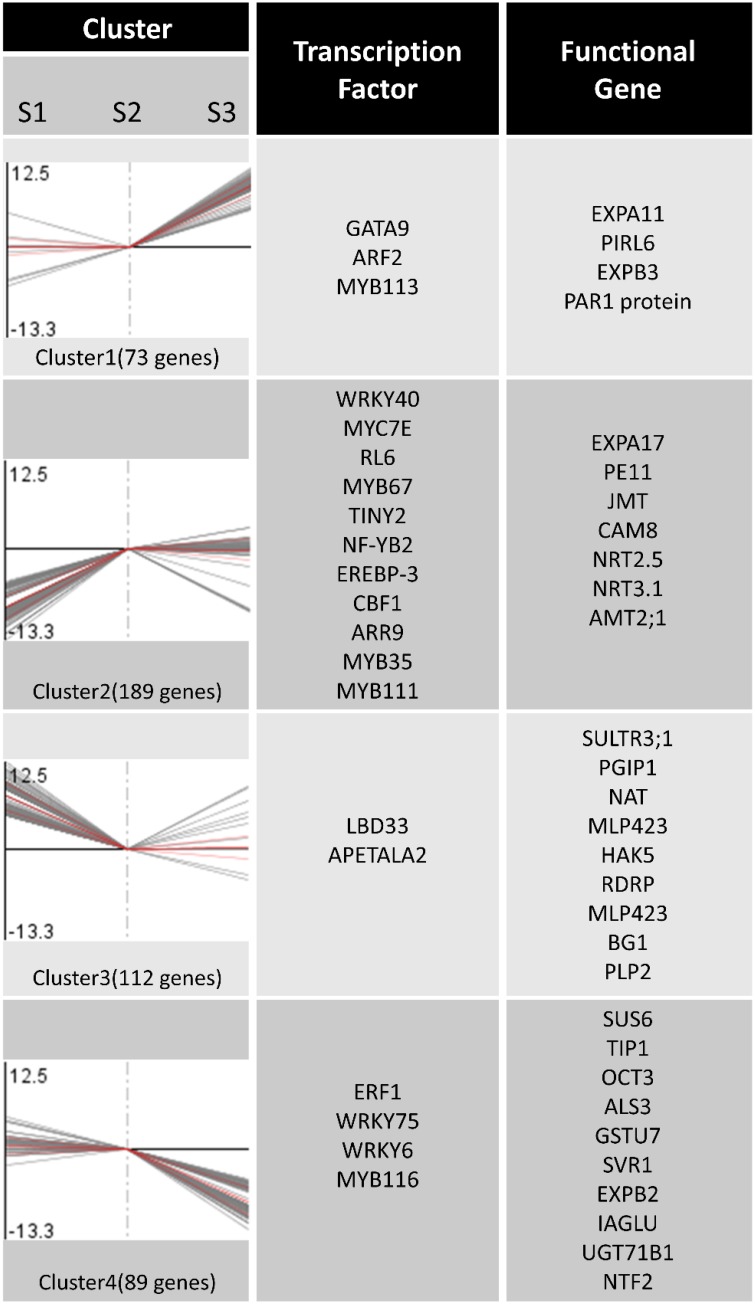
**Expression patterns clustering and gene annotation**. Two-dimensional hierarchical clustering classifies 463 differential gene expression profiles into four expression cluster groups according to the similarity of their expression profiles.

Based on a similarity of gene expression profiles of two-dimensional hierarchical clustering, we classified 463 differential expression profiles into four expression cluster groups (Clusters 1, 2, 3, and 4; Figure 4). Visual inspection of these expression groups suggested diverse and complex patterns of gene regulation. Clusters 1 and 4 contained the genes induced or repressed by ${\text{NO}}_{3}^{-}$, while the genes induced or repressed by ${\text{NH}}_{4}^{+}$ were grouped in Cluster 2 and 3 (Figure 4).

**Figure 5:**
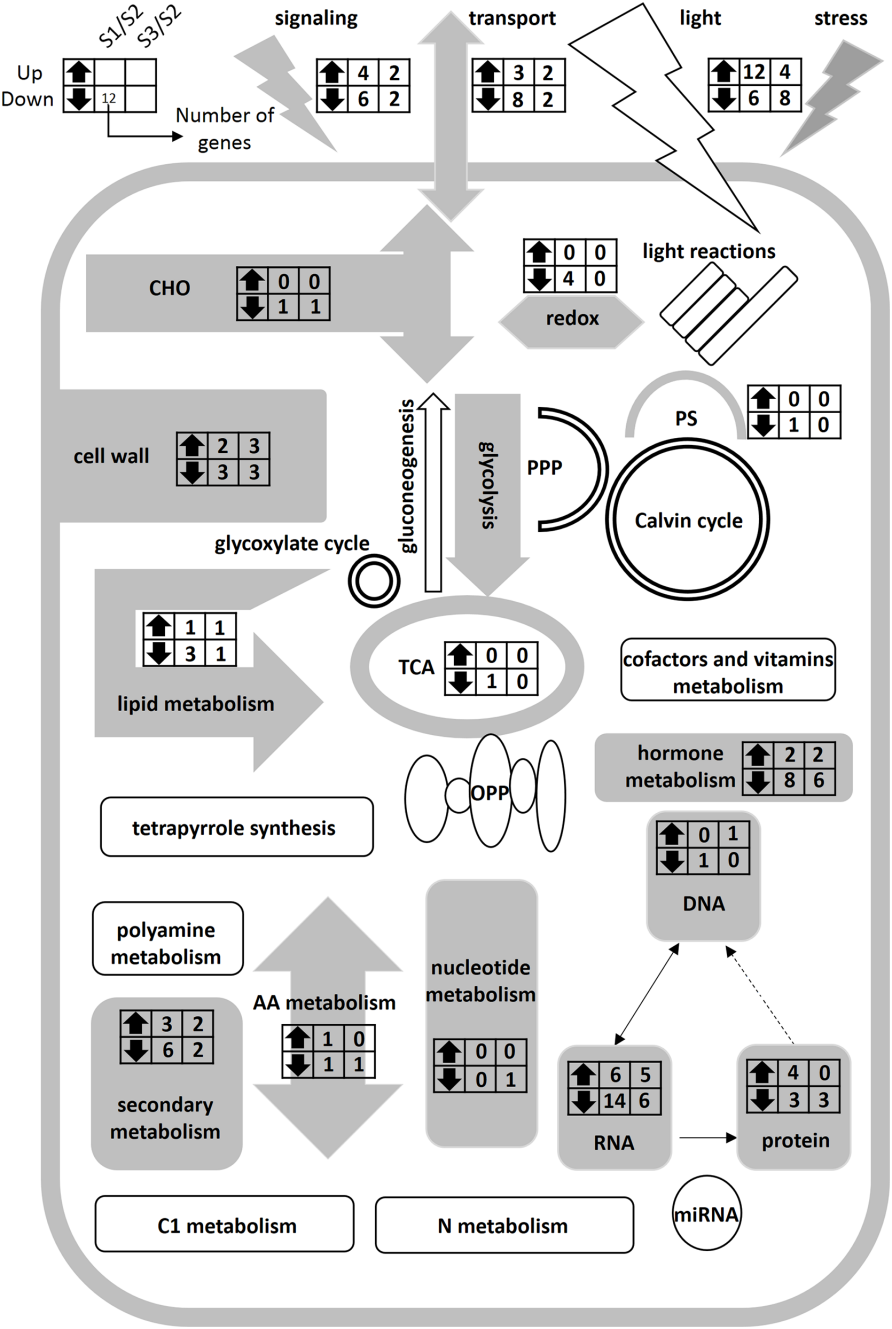
**Schematic representation of primary metabolism of poplar roots at different forms of nitrogen**. Gray charts represent significantly differentially expressed genes in the metabolic pathways. White charts represent the genes without significantly different expression in the metabolic pathways. The left and right columns represent the S1/S2 and S3/S2 at the transcriptional level, respectively. Up- and down-facing triangles represent an increase and decrease in transcripts. The digital in columns represent the number of change genes. It can be found that there was no significantly difference gene in nitrogen metabolic pathway.

In order to identify differentially expressed genes and transcription factors that are associated with different N forms, we identified 20 putative TF genes through the Mapman Transcription factors Database (pathway). Meanwhile, 30 functional genes associated with N forms are shown in Figure 4; the entire data were listed in Table S5. Figure 4 showed that the expression level of extension of cell wall related genes such as EXPA11 and EXPB3 (Cluster 1) were significantly increased under ${\text{NO}}_{3}^{-}$ treatment. In Cluster 2, the genes encoding ${\text{NH}}_{4}^{+}$ transporters and ${\text{NO}}_{3}^{-}$ transporters (such as AMT2;1, NRT 2.5, NRT3.1) were changed significantly under ${\text{NO}}_{3}^{-}$ and NH_4_NO_3_ forms. Under ${\text{NH}}_{4}^{+}$ treatment, the expression levels of the genes encoding N storage proteins are significantly increased (PLP2). Interestingly, there was no significant difference in genes expression in N metabolism pathway, mitochondrial electron transport/ATP synthesis and mineral nutrition (Figure 5; Figure S3).

## Discussion

In this study, we aimed to investigate transcriptional-level changes of poplar roots under different N forms (${\text{NH}}_{4}^{+}$, ${\text{NO}}_{3}^{-}$, and NH_4_NO_3_) for a long time (21 days). We found root length and dry weight in ${\text{NO}}_{3}^{-}$ or NH_4_NO_3_ significantly higher than that in ${\text{NH}}_{4}^{+}$ condition. Our results (Figure 2) are in agreement with that found in the rice (*Oryza Sativa L*.; Schortemeyer et al., 1997; Li et al., 2006). To better understand the changes of root morphology and growth under different N forms, we examined differentially expressed genes of poplar roots treated by different N forms (Tables S2–S4). Based on the result of GO analysis, we only found enrichment of GO term oxidative stress under ${\text{NH}}_{4}^{+}$ treatment and GO term cell wall under ${\text{NO}}_{3}^{-}$ treatment. Podgorska et al. (2013) analyzed physiological responses (up to 8 weeks) of *Arabidopsis* leaves to ${\text{NO}}_{3}^{-}$ or ${\text{NH}}_{4}^{+}$ treatment and found that ${\text{NH}}_{4}^{+}$ nutrition led to increase of leaf NAD(P)H/NAD(P)^+^ ratio, reactive oxygen species content and accumulation of biomolecules oxidized by free radicals of *Arabidopsis thaliana*. Patterson et al. (2010) believed that the ${\text{NH}}_{4}^{+}$-induced responses were primarily associated with biotic stress and cellular redox (Patterson et al., 2010). In this paper, our results are consistent with the above results.

We examined genes expression of N-form associated metabolic pathways, but did not find significantly differentially expressed genes in N metabolism pathway. In a previous study on transcript levels of barley plants supplied with ${\text{NO}}_{3}^{-}$, ${\text{NH}}_{4}^{+}$, or NH_4_NO_3_ for 48 h, only three genes were found to be specifically ${\text{NO}}_{3}^{-}$/${\text{NH}}_{4}^{+}$-induced/repressed (Lopes and Araus, 2008). Hoffmann et al. (2007) studied the genes expression of *Arabidopsis* seedlings continuously on the medium containing ${\text{NH}}_{4}^{+}$ or ${\text{NO}}_{3}^{-}$ for 15 days, and only found two genes differentially expressed under different N forms. In the study of Beatty et al. (2009), the alanine aminotransferase (AlaAT) gene was transferred into rice plants and over-expressed through a tissue-specific promoter; the authors found the transgenic plants had a strong N use efficiency but less change in the transgenic transcriptome as compared with the controls, with only 0.11 and 0.07% differentially regulated genes in roots and shoots, respectively. According to significant difference of N transport-related genes (Figure 5), we assumed that N transport related genes play an important role in the regulation of long-time N uptake, and N metabolism related genes may be in a steady state in the poplar roots under N forms treatment for 21 days.

In this study, we analyzed genes expression of N-form treated poplar roots by Mapman software (Thimm et al., 2004); and identified four gene expression clusters, a ${\text{NO}}_{3}^{-}$-induced cluster (Cluster 1), an ${\text{NH}}_{4}^{+}$-repressed cluster (Cluster 2), an ${\text{NH}}_{4}^{+}$-induced cluster (Cluster 3), and a ${\text{NO}}_{3}^{-}$-repressed cluster (Cluster 4), respectively. The significantly differentially expressed genes (Table S5) were classified to 20 metabolism pathways (Figure 5), some of which (30 functional genes) have been clearly annotated in Figure 4. We found gene families members related to the synthesis of cell wall in different clusters (Figure 4; Table S5). For example, *exp3* and *expA11* belong to cluster 1, and *expA17* belongs to cluster 2, while paralog *expB2* belongs to cluster 4 (Figure 4). EXPA and EXPB were known to have cell-wall loosening activity and to be involved in cell expansion and other developmental events during which cell-wall modification occurs (Cosgrove, 2000). EXPA and several EXPB are implicated as catalysts of “acid growth,” and regulate the expansion activity rapidly by modulating pH of cell wall (McQueen-Mason et al., 1992; Li et al., 1993, 2003; Cho and Kende, 1997; Sampedro and Cosgrove, 2005). In fine roots of *Populus popularis*, net fluxes of ${\text{NH}}_{4}^{+}$ and ${\text{NO}}_{3}^{-}$ are in association with H^+^ fluxes and change the pH around the root (Luo et al., 2013b). In the present study, the transcriptional level changes of *exp* genes may be related only to N form but not to change of extracellular pH; because we replaced the culture solution per 2 days for eliminating the effect of medium pH. It is inferred that the *exp* family genes may play a key role in morphogenesis of the poplar roots when they are treated by different N forms for a long time.

Nitrogen absorption related genes, for example, *NRT2.5* and *NRT3.1*, and the genes associated with ${\text{NH}}_{4}^{+}$ absorption, *AMT2.1*, occurred in the cluster 2. The *NRT3* family in *Arabidopsis* contained two members, *AtNRT3.1* and *AtNRT3.2*. The *NRT3* family genes in *Arabidopsis* play a role in ${\text{NO}}_{3}^{-}$ transport (Okamoto et al., 2006; Orsel et al., 2006). The two *NRT3* genes appear to be closely correlated with each other, but *NRT3.1* (*NAR2.1*) appears to play a more significant role in high-affinity ${\text{NO}}_{3}^{-}$ uptake (Okamoto et al., 2006). These genes are not ${\text{NO}}_{3}^{-}$ transporters, but have been shown necessary for ${\text{NO}}_{3}^{-}$ transport through interaction with the other *NRT2* transporters (Plett et al., 2010). From Figure 4, we found that the expression of *NRT3.1* gene was inhibited only by ${\text{NH}}_{4}^{+}$ treatment, whereas under ${\text{NO}}_{3}^{-}$ or NH_4_NO_3_ treatment, *NRT3.1* expression level was increased significantly as compared with that with ${\text{NH}}_{4}^{+}$ treatment. So we considered that ${\text{NO}}_{3}^{-}$ might be an essential for *NRT3.1* expression, which would promote N absorption. *AMT2.1* genes that are associated with ${\text{NH}}_{4}^{+}$ absorption were expressed in low abundance of ${\text{NH}}_{4}^{+}$ as a sole nitrogen source.

Poplar *BSP* genes, which belong to the nucleoside phosphorylases gene family, are expressed before the metabolic nucleotide salvaging, and play an important role in ecophysiological adaptation for inter- and intra-seasonal N storage and cycling (Pettengill et al., 2013). In this study, we obtained a transcription read of *bsp-like* gene (Comments as PLP; Figure 4), which might be a kind of storage protein of a poplar, specifically induced by ${\text{NH}}_{4}^{+}$. The magnitude of gene expression in stems is significantly higher than that of leaves and roots, and has a high homology with the NP-like subfamily of the BSP gene family (data not shown). We speculated that the *BSP* protein synthesis may have a close correlation with the exogenous ${\text{NH}}_{4}^{+}$ concentration.

Recent publications show that *myb48* (Plavcova et al., 2013) and *wrky75* (Devaiah et al., 2007) are significantly differentially expressed under different N treatments. In the present study, we identified 20 transcription factors (TF) mRNAs that have close correlations with N forms. However, potential roles of the identified TF mRNAs in root morphogenesis are unknown. So functional verification of the transcription factors is necessary in future work.

In summary, there are significant differences in morphological characteristics and N transport-related genes of the poplar roots between the three N forms for 21 days, but with no significant difference in the transcription level in N-metabolism pathway (Figure 5). Further study should focus on transcriptional level changes of poplar roots treated by different N forms for a short-term period using RNA-SEQ and reveal potential molecular mechanisms. Further work is proposed to select functional genes related to root morphology and functional assignment of the transcription factors under different nitrogen forms.

## Author contributions

Conceived and designed the experiments: CQ, GL. Performed the experiments: CQ, ZX. Analyzed the data: CQ, ZX, CY, YL. Revised the paper: YH, GL, GS. Wrote the paper: CQ, GL. All authors have read and approve of the final manuscript.

### Conflict of interest statement

The authors declare that the research was conducted in the absence of any commercial or financial relationships that could be construed as a potential conflict of interest.

## Supplementary material

Table S1 **The genes primers used for qRT-PCR validation are listed**.

Table S2–S4 **To determine which of the 40,361 genes were differentially expressed among the different N-treatments libraries, we required a two-fold or greater change in expression and Probability ≥ 0.8, which resulted in a set of 602 DEGs**.

Table S5 **The annotation results of expression pattern clustering analysis**.

Figure S1 **Morphological parameters of poplar roots under different N forms for 21 days. Upper:** a whole seedling under different N forms, respectively; **Lower:** a detailed root structure. Table- dry root weights. Values are the mean of four replicates ± SE. Different letters mean significant difference at a significance of 0.05 through ANOVA analysis and Duncan's test.

Figure S2 **Validation of RNA-SEQ results by real-time PCR; r is the correlation coefficient value between the two platforms**.

Figure S3 **Alidation of nitrogen metabolism-related genes expression by real-time PCR**.
