# Supplementary figures and images for: RNA-SEQ Reveals Transcriptional Level Changes of Poplar Roots in Different Forms of Nitrogen Treatments
Source: Front Plant Sci. 2016 Feb 2;7:51. doi: 10.3389/fpls.2016.00051 (PMC4735414; doi:10.3389/fpls.2016.00051)

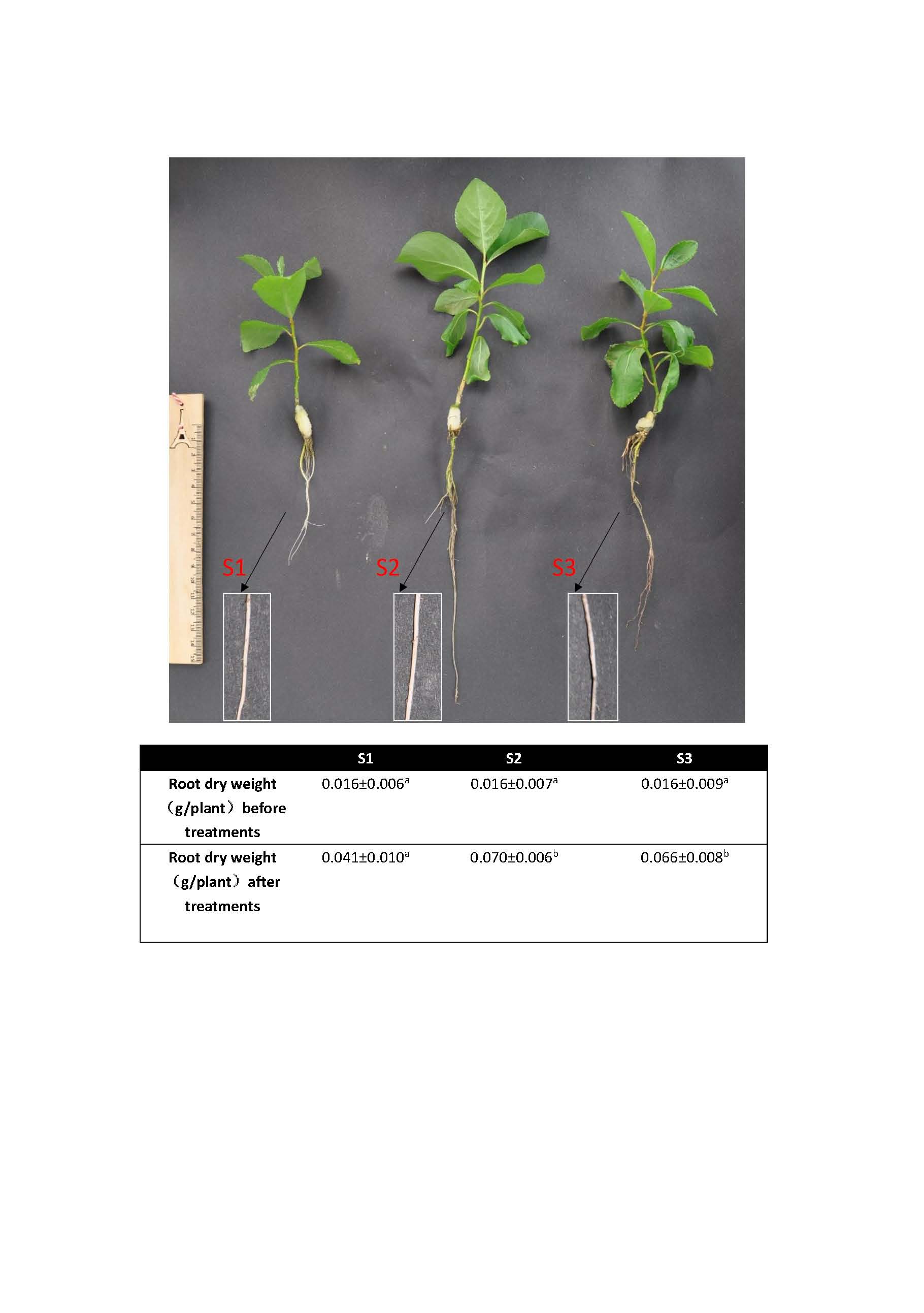

Supplement: Figure S1 — Morphological parameters of poplar roots under different N forms for 21 days. Upper: a whole seedling under different N forms, respectively; Lower: a detailed root structure. Table- dry root weights. Values are the mean of four replicates ± SE. Different letters mean significant difference at a significance of 0.05 through ANOVA analysis and Duncan's test. [file Image1.JPEG]

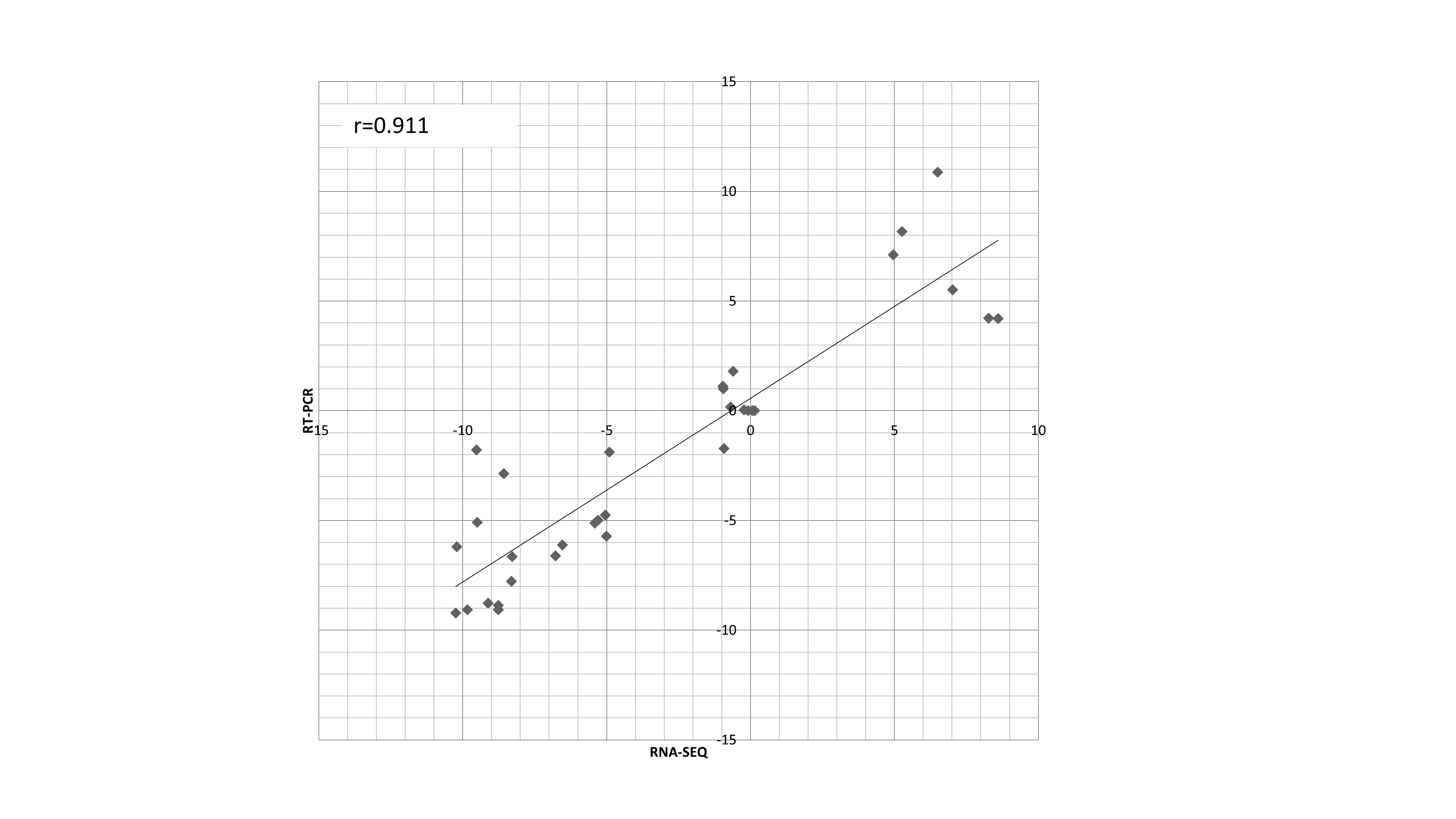

Supplement: Figure S2 — Validation of RNA-SEQ results by real-time PCR; r is the correlation coefficient value between the two platforms. [file Image2.JPEG]

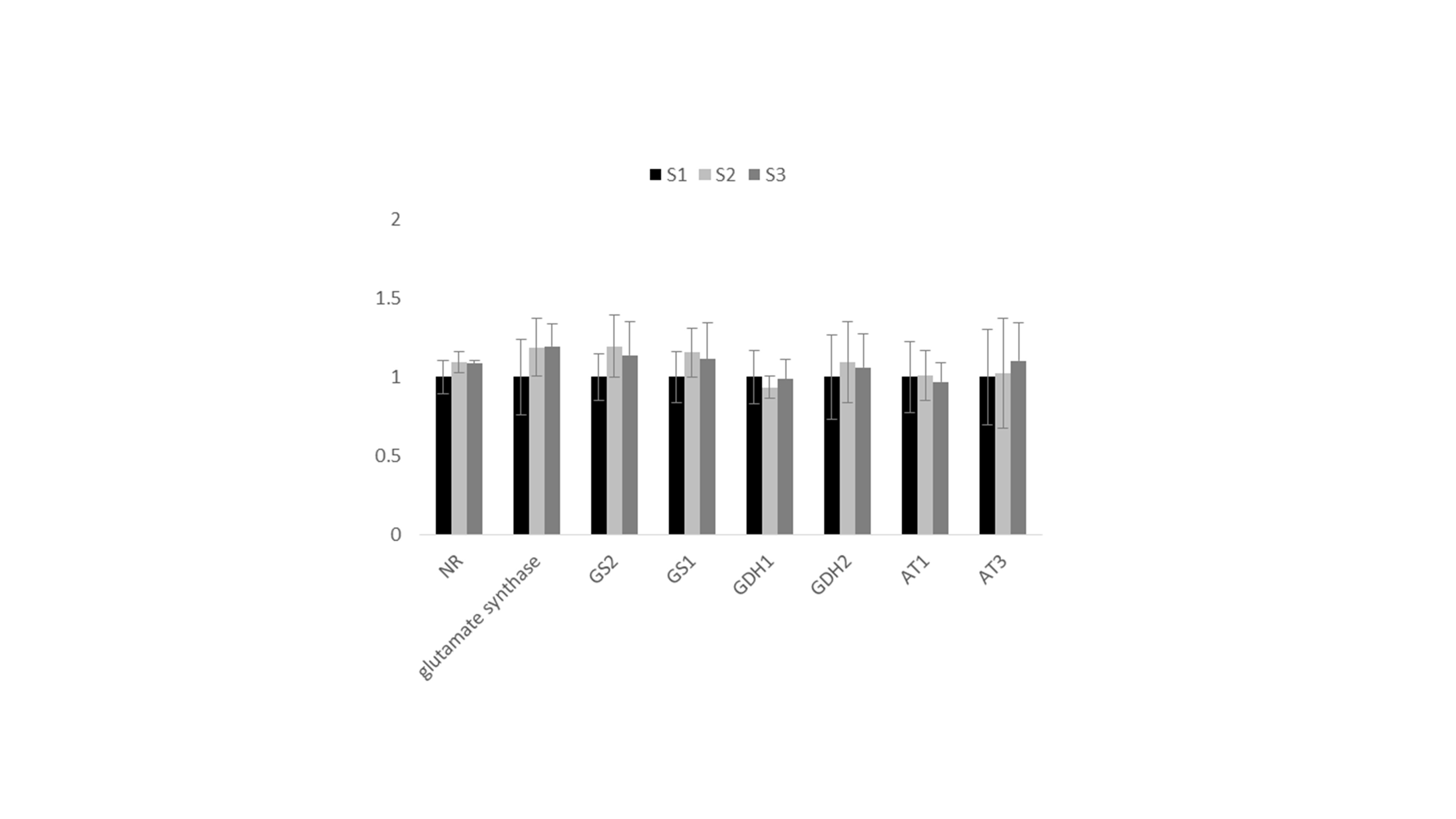

Supplement: Figure S3 — Alidation of nitrogen metabolism-related genes expression by real-time PCR. [file Image3.JPEG]
